# Closure rates and patterns after light silicone oil tamponade for persistent full-thickness macular holes

**DOI:** 10.1007/s00417-023-06215-w

**Published:** 2023-08-29

**Authors:** Felix Innauer, Maximilian Gabriel, Christoph Mayer-Xanthaki, Anton Haas

**Affiliations:** Department of Ophthalmology, Medical University of Graz, Auenbruggerplatz 4, 8036 Graz, Austria

**Keywords:** Pars plana vitrectomy, Persistent macular hole, Refractory macular hole, Silicone oil

## Abstract

**Purpose:**

To report outcomes of re-vitrectomy using light silicone oil (SO) tamponade for persistent macular holes (MHs).

**Methods:**

We reviewed cases of patients with full-thickness MHs that underwent pars plana vitrectomy (PPV) with air/gas and were re-vitrectomized using light SO tamponade after primary non-closure (persistent MHs). Outcome measures included anatomic closure rates and patterns confirmed by optical coherence tomography (OCT) and changes in best-corrected visual acuity (BCVA).

**Results:**

A total of 42 eyes of 41 patients with unsuccessful primary PPV with air/gas were included. After re-vitrectomy with light SO (1000-centistoke), 29 (69%) eyes demonstrated type 1 closure without neurosensory defects in OCT scans, whereas 9 eyes (21%) showed type 2 closure with persisting neurosensory defects. Available data (*n* = 21) showed a significant mean improvement of BCVA from 0.99 logMAR (SD 0.25, range 0.7–1.3) preoperatively to 0.74 logMAR (SD 0.42, range 0.2–1.5) postoperatively (*p* = 0.035).

**Conclusion:**

The treatment of persistent MHs with PPV and light SO tamponade resulted in high closure rates.

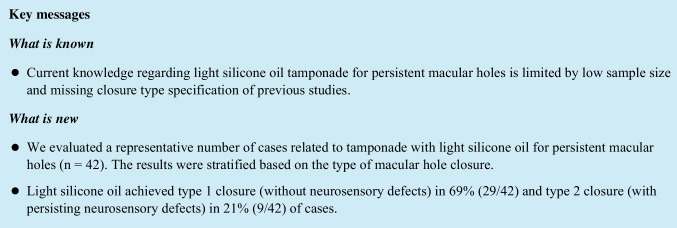

## Introduction

Full-thickness macular holes (MHs) constitute structural breaks in the fovea, resulting in significant loss of central vision and metamorphopsia. This condition has been treated using pars plana vitrectomy (PPV) with air/gas for decades [1], but refractory macular holes remain a surgical challenge to this day [2, 3]. These complicated cases usually present with large preoperative diameters [2]. Per definition, persistent MHs fail to close after primary surgery, whereas recurrent MHs reopen at least 4 weeks after primary surgery [4]. Both conditions require a second intervention, although it is unclear which surgical approach offers the best outcomes [2]. Options include implanting a scaffold for glial proliferation (internal limiting membrane (ILM) translocation/insertion/free flap, amniotic membrane, lens capsule, retinal tissue), increasing retinal compliance (macular hole hydrodissection/induction of retinal detachment), stimulation of retinal adhesion (autologous blood, autologous platelet concentrate), and the instillation of long-acting tamponades such as silicone oil (SO) [5]. According to modern OCT-based classifications, there are three different surgical endpoints after macular hole surgery: elevated open (non-closure; edges of the hole elevated and visible in funduscopy), flat open (type 2 closure; edges of the hole flat and visible), and flat closed (type 1 closure; edges of the hole flat and not visible), with the latter offering the best visual outcomes [6, 7]. A recent meta-analysis found that the mean closure rate of all surgical techniques was 78% for persistent and 80% for recurrent MHs, although high levels of vision were uncommon [8].

Historically, SO tamponade was also utilized in primary macular hole surgery [9] before it was replaced with air (and later gas) tamponade [1]. Eventually, it was rediscovered for the treatment of refractory macular holes and promising closure rates were reported, although these investigations were limited by low sample sizes, missing disclosure of anatomical closure patterns and the usage of different SO types in different studies [10–14]. A prospective randomized study previously showed that heavy SO was more efficacious than gas in closing persistent MHs [14]. On the other hand, studies investigating conventional gas refill reported similar closure rates to SO instillation [15]. With this investigation, we want to provide a thorough analysis of real-world cases treated with light SO tamponade after unsuccessful primary macular hole surgery. Special attention has been dedicated to OCT-based macular hole closure types, as these patterns determine visual outcomes and have seldom been investigated in previous studies.

## Materials and methods

### Study population

We retrospectively reviewed the medical records of patients operated for persistent macular holes using SO tamponade between 2007 and 2022 at the Department of Ophthalmology of the Medical University Graz, Austria. All data were collected from a customized in-house documentation database. Patients were included if they were operated for persistent macular holes using SO tamponade during the study period and if OCT scans were available before initial surgery, before SO tamponade, and after SO instillation. Cases were excluded if OCT scans were unavailable or of insufficient image quality. Eyes that had degenerative myopia or an axial length greater than 26 mm were excluded. Other secondary MHs and concomitant diseases did not constitute a reason for exclusion. MH size was measured using the minimum linear diameter [16].

### Surgery

Primary vitrectomy was performed by one of seven experienced retinal surgeons using 23-, 25-, or 27-gauge ports according to the surgeon’s preference. Sclerotomies were located superonasally, superotemporally, and inferotemporally (infusion line) at a distance of 3.5 mm to the limbus. The internal limiting membrane was routinely stained with a combination of 0.025% Brilliant Blue G and 0.15% Trypan Blue (Membrane Blue-Dual, DORC, Zuidland, the Netherlands) and then grasped near the superior or inferior arcades, resulting in an ILM peeling size of approximately two disc diameters centered on the foveola. Additional cataract surgery was performed and ILM flaps were created according to the individual assessment of the surgeon. Air or SF6 were used as tamponade. Standard postoperative therapy consisted of topical neomycinesulfate and betamethasonedinatrium-phosphate. All patients were advised to adhere to postoperative face-down positioning for 2–3 days. Re-vitrectomy with SO was again performed by one of seven experienced retinal surgeons, and 20-, 23-, 25-, or 27-gauge ports were used according to the surgeon’s preference. 1000-centistoke SO was used as tamponade. Standard postoperative therapy was identical to primary surgery and all patients were again advised to adhere to postoperative face-down positioning for 2–3 days.

### Outcome measures and statistical analysis

The MH closure rate after SO tamponade served as the main outcome measure. Changes in best-corrected visual acuity (BCVA, logMAR) before and after SO tamponade served as the secondary outcome measure. BCVA changes were analyzed in patients who had BCVA measurements at least 14 days after SO removal or after SO instillation if SO was left in situ indefinitely.

Metric parameters were descriptively summarized using mean, standard deviation and range (minimum–maximum), or median and range. Categorical parameters were given as absolute and relative frequencies. Microsoft Excel 2019 (Microsoft Corporation, Redmond, USA) was used for data management. Calculations were performed using IBM SPSS Statistics (Version 27, 2020, International Business Machines Corporation, Armonk, NY, USA). The Shapiro-Wilk test was used to assess if continuous variables followed a normal distribution. The paired Student’s *t*-test was used to assess statistical significance for normally distributed variables. For non-normally distributed variables, statistical significance between two or more groups was assessed using the Kruskal-Wallis test. All analyses are regarded exploratory in nature and a *p*-value of < 0.05 was considered significant.

## Results

Forty-eight eyes were re-operated using light silicone oil during the study period and were therefore reviewed. After excluding three eyes due to missing OCT images and three eyes due to degenerative myopia and an axial length greater than 26 mm, 42 eyes of 41 patients were eventually analyzed. Mean patient age at the time of SO instillation was 68 years (SD 11, range 28–84), 64% of patients were female and 50% were right eyes (Table 1). Before initial surgery, the mean axial eye length was 23.2 mm (*n* = 39; SD 0.9, range 21.6–25.4) and the mean spherical equivalent was 0.17 diopters (SD 1.89, range − 6–4.25). Medical chart review revealed additional epiretinal membranes in 8 eyes, high myopia in 2 eyes (both − 6 diopters) and vitreous hemorrhage and branch retinal vein occlusion in one patient each before primary surgery. Seven eyes (17%) had small (mean minimum linear diameter 164 μm, SD 60, range 94–247), 18 (43%) medium (323 μm, SD 32, range 259–370), and 17 (40%) large (520 μm, SD 72, range 411–660) MHs before initial PPV according to the International Vitreomacular Traction Study Group [17]. Total mean macular hole minimum linear diameter was 376 μm (SD 143, range 94–660). Cystoid rim (intraretinal cysts around the edges of MHs) and fluid cuff (subretinal fluid) [18] in initial OCTs were found in 40 (95%) and 17 (40%) eyes, respectively.

**Table 1:** Descriptive statistics of patients with macular holes, stratified by macular hole closure type after silicone oil tamponade

	Total (*n* = 42)	Type 1 closure (*n* = 29)	Type 2 closure (*n* = 9)	Nonclosure (*n* = 4)
Age (years)	68 ± 11 (28–84)	69 ± 9 (50–84)	63 ± 15 (28–78)	67 ± 13 (49–79)
Female	27/42 (64)	20/29 (69)	5/9 (55)	2/4 (50)
Male	15/42 (36)	9/29 (31)	4/9 (45)	2/4 (50)
MH diameter^a,b^ (μm)	484 ± 164 (136–833)	467 ± 164 (136–833)	500 ± 108 (278–658)	566 ± 267 (189–817)
Small MH^b,c^	2/42 (5)	1/29 (3)	0/9 (0)	1/4 (25)
Medium MH^b,c^	9/42 (21)	8/29 (28)	1/9 (11)	0/4 (0)
Large MH^b,c^	31/42 (74)	20/29 (69)	8/9 (89)	3/4 (75)
MH > 650 μm^b,d^	6/42 (14)	4/29 (14)	1/9 (11)	1/4 (25)
Axial eye length (mm, *n* = 39)	23.2 ± 0.9 (21.6–25.4)	23.1 ± 0.9 (21.6–24.7)	23 ± 1 (21.8–24.1)	24.2 ± 0.9 (23.3–25.4)
Cystoid rim^b^	40/42 (95)	29/29 (100)	7/9 (78)	4/4 (100)
Fluid cuff^b^	38/42 (90)	27/29 (93)	8/9 (89)	3/4 (75)
Pseudophakic eyes^b^	32/42 (76)	23/29 (79)	7/9 (78)	2/4 (50)
Cataract surgery during SO installation	6/42 (14)	5/29 (17)	0/9 (0)	1/4 (25)
Weeks between first consultation and primary surgery, median (range)	2.7 (0–26.9)	2.7 (0–26.9)	2.6 (0.1–8)	2.4 (1.6–9.7)
Weeks between primary surgery and SO installation, median (range)	11.6 (1–91.1)	11.3 (2.6–91.1)	13.7 (1–25)	11.1 (7.3–16.7)
Weeks of SO tamponade, median (range)	16 (8–46.7)	15.8 (8–46.7)	17.7 (12.6–23.3)	16.4 (11–25.4)
BCVA before primary PPV (logMAR)	0.77 ± 0.24 (0.4–1.3) (*n* = 21)	0.8 ± 0.2 (0.6–1.3) (*n* = 14)	0.97 ± 0.35 (0.6–1.3) (*n* = 3)	0.53 ± 0.1 (0.4–0.6) (*n* = 4)
BCVA before SO installation (logMAR)	0.99 ± 0.25 (0.7–1.3) (*n* = 21)	0.97 ± 0.25 (0.7–1.3) (*n* = 14)	0.97 ± 0.29 (0.8–1.3) (*n* = 3)	1.1 ± 0.3 (0.7–1.3) (*n* = 4)
BCVA after SO installation (logMAR)	0.74 ± 0.42 (0.2–1.5) (*n* = 21)	0.53 ± 0.28 (0.2–1.1) (*n* = 14)	1.1 ± 0.53 (0.5–1.5) (*n* = 3)	1.2 ± 0.2 (1–1.3) (*n* = 4)

Data are presented as either mean ± standard deviation (range), median (range), or *x*/*n* (%). Percentages refer to available observations. (*SO* silicone oil, *MH* macular hole, *BCVA* best-corrected visual acuity)

^a^Minimum linear diameter [16]

^b^Before SO tamponade

^c^According to International Vitreomacular Traction Study: small ≤ 250 μm, medium 251–400 μm, large > 400 μm [17]

^d^According to Manchester Large Macular Hole Study: large > 650 μm [3]

### Initial surgery

Median time between the patient’s first consultation at our department and initial PPV was 2.7 weeks (range 0–26.9). Twenty-three-, 25-, and 27-gauge vitrectomy were used in 27 (64%), one (3%), and six (14%) eyes, respectively. In eight eyes (19%), port size was not specified in the surgical report. SF6 and air tamponade were used in 36 (86%) and 6 (14%) eyes, respectively.

Epiretinal membranes were peeled in 8/42 cases (19%). The internal limiting membrane was initially peeled in 39 eyes (93%). ILM flaps were created in 3 (7%) patients during initial surgery. Four eyes were pseudophakic (10%) before initial MH surgery and 28 phakic patients received combined phacovitrectomy, resulting in 32 (76%) pseudophakic patients after initial surgery. All patients were advised to adhere to postoperative face down positioning. After initial surgery, the mean macular hole minimum linear diameter increased significantly from 376 μm (SD 143, range 94–660) at baseline to 484 μm (SD 164, range 136–833) (*p* < 0.001, paired *t*-test).

### Secondary surgery using silicone oil tamponade

Median time between the initial surgery and SO surgery was 11.6 weeks (range 1–91.1). Two eyes (5%) had small (mean minimum linear diameter 163 μm, SD 37, range 136–189), nine (21%) medium (314 μm, SD 30, range 278–370), and 31 (74%) large (554 μm, SD 125, range 402–833) MHs before SO instillation according to the International Vitreomacular Traction Study Group [17]. Total mean macular hole minimum linear diameter was 484 μm (SD 164, range 136–833) before SO. Cystoid rim and fluid cuff were found in 40 (95%) and 38 (90%) eyes, respectively.

Re-vitrectomy with light SO was the first choice for re-operation in all cases. Twenty-, 23-, 25-, and 27-gauge vitrectomy were used in one (2%), 29 (69%), three (7%), and four (10%) eyes, respectively. In five eyes (12%), port size was not specified in the surgical report. Thirty-two eyes were pseudophakic (76%) before SO instillation and six phakic patients received combined phacovitrectomy, resulting in 38 (90%) pseudophakic patients after SO surgery. 1000-centistoke SO was used in all eyes. Nine eyes (21%) received additional peeling of ILM remnants. Additional ILM flaps were created in three eyes. The median duration of silicone oil tamponade was 16 weeks (range 8–46.7). Ninety-five percent (95%, 40/42) of patients were pseudophakic after two patients received combined phacoemulsification during silicone oil removal. One patient (2%) refused SO removal. For 21 eyes (including the patient refusing SO removal), OCT scans were only available before SO removal. For 21 eyes, OCT scans were also available after SO removal.

**Fig. 1:**
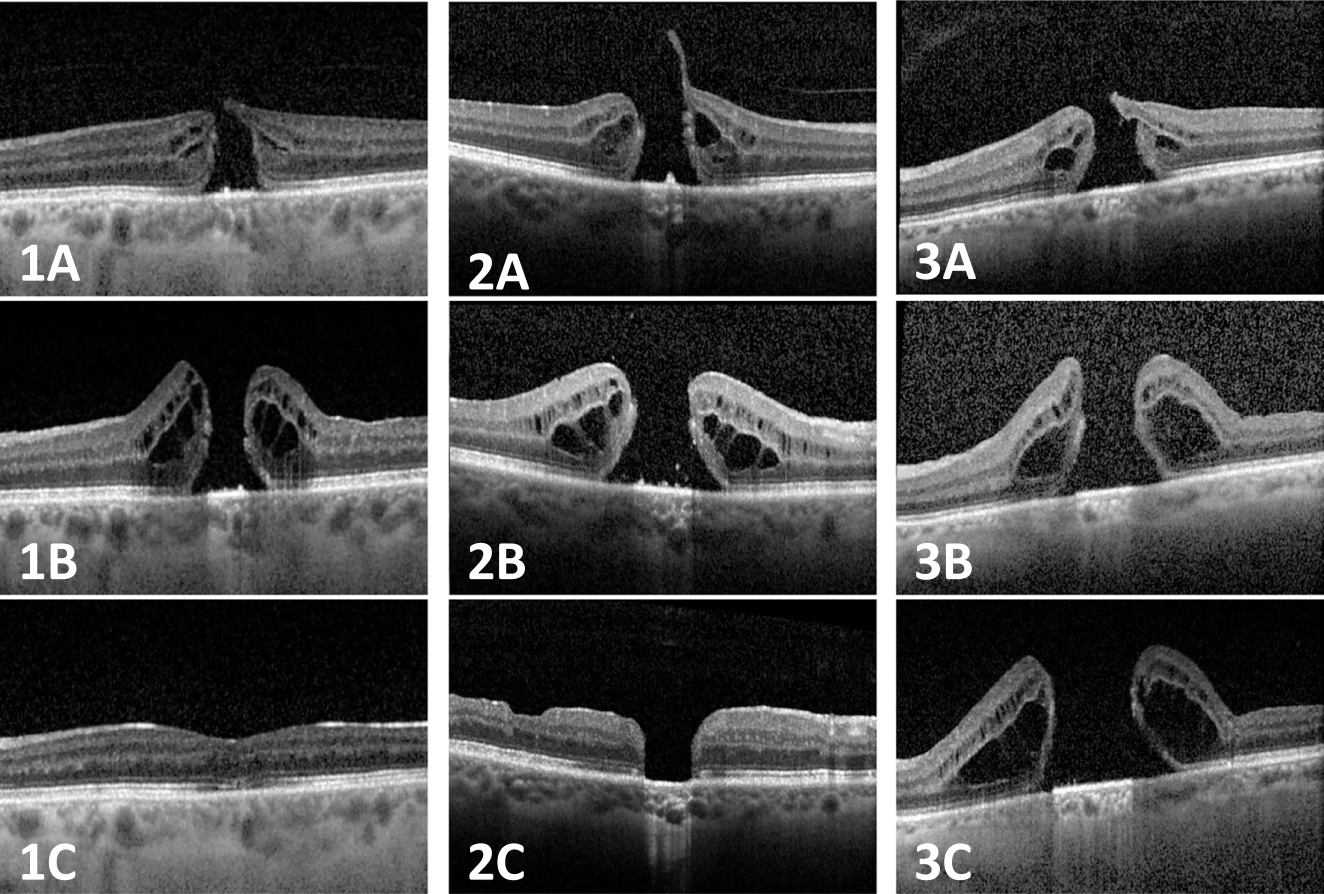
OCT image history of three patients (1–3): macular holes before primary surgery (1A, 2A, 3A). Persistent macular holes before silicone oil tamponade (1B, 2B, 3B). Endpoints: flat closed (type 1 closure) (1C), flat open (type 2 closure) (2C), elevated open (no closure) (3C)

Type 1 (flat closed) and type 2 closure (flat open) were achieved in 29 (69%) and 9 eyes (21%), respectively (see Table 1 and Fig. 1). BCVA change (preoperative–postoperative) was calculated using available data (*n* = 21). Mean BCVA improved significantly from 0.99 logMAR before SO instillation to 0.74 logMAR postoperatively (*p* = 0.035, paired *t*-test). After SO surgery, type 1 closure resulted in a significant improvement in mean BCVA from 0.97 to 0.53 logMAR (*p* = 0.02, paired *t*-test, *n* = 14). The visual acuity of eyes with type 2 closure (*n* = 3, BCVA change from 0.97 logMAR preoperatively to 1.1 logMAR postoperatively) and elevated open configuration (*n* = 4, BCVA change from 1.1 logMAR preoperatively to 1.2 logMAR postoperatively) did not improve notably. The aforementioned eyes that received additional ILM flaps (*n* = 3) achieved flat closed (type 1 closure, *n* = 2) and flat open (type 2 closure, *n* = 1) configurations. The time interval between initial vitrectomy and SO instillation had no significant influence on the anatomic closure pattern (*p* = 0.485, Kruskal-Wallis). Postoperative complications after SO instillation and removal included cystoid macular edema (*n* = 3) and retinal detachment (*n* = 1). Mean intraocular pressure (IOP) was 14.5 mmHg (n = 39, SD 2.8, range 10–21) 1 day before SO instillation and 14.1 mmHg (*n* = 39, SD 4.9, range 1–25) after SO instillation. The first postoperative IOP measurement was taken at a median of 3 days after SO instillation (range 1–142). Only five eyes had an IOP elevation > 21 mmHg during SO tamponade, with peak values of 22, 24, 25, 30, and 34 mmHg, respectively. Two patients required topical antiglaucoma therapy and one of them also received a single dose of oral acetazolamide. The IOP was lowered adequately in both cases. Two eyes had postoperative hypotony, which resolved spontaneously.

### Unclosed macular holes

#### Elevated open configuration

The elevated open configuration was observed in four cases after re-vitrectomy. The median time interval between the first consultation at our department and initial surgery was 2.4 weeks (range 1.6–9.7) and 11.1 weeks (range 7.3–16.7) between primary surgery and SO instillation. Of these four cases, three remained open after SO instillation and one reopened 5 months after SO removal. One unclosed MH was small (minimum linear diameter 189 μm) and three were large (mean minimum linear diameter 568 μm, SD 219, range 616–817). Cystoid rim and fluid cuff were found in four and three eyes, respectively. SO was left in situ for a median time of 16.4 weeks (range 11–25.4). One MH was finally successfully closed after SO re-instillation.

#### Flat open configuration

Out of nine MHs with a final flat open configuration, one was of medium (minimum linear diameter 278 μm) and eight were of large size (mean minimum linear diameter 528 μm, SD 74, range 443–658). Cystoid rim and fluid cuff were present in seven and eight eyes, respectively. The median time intervals between the first consultation at our department and initial surgery were 2.6 weeks (range 0.1–8), from initial surgery to SO instillation 13.7 weeks (range 1–25), and from SO instillation to SO removal 17.7 weeks (range 12.6–23.3), respectively.

## Discussion

Pars plana vitrectomy, ILM peeling and gas tamponade are widely accepted as the initial treatment of choice for full-thickness macular holes. However, it is still unclear which surgical technique is the most effective after primary non-closure. We reviewed the medical records of cases with persistent full-thickness macular holes and were able to determine closure rates and patterns using light SO tamponade. Anatomic type 1 (flat closed configuration) closure was achieved in 69% (29/42) of cases. In nine (21%) and four cases (10%), anatomic closure was not achieved (flat open configuration and elevated open configuration). This study represents the series with the highest number of cases (*n* = 42) treated with light (1000-centistoke) SO.

Different groups have previously investigated the performance of SO tamponade in persistent/recurrent macular holes [11–14, 19–21]. Schaub et al. reported a closure rate of 45.7% using heavy silicone oil in 35 persistent macular holes (mean minimum linear diameter 518 µm ± 171.1) without specifying closure patterns [22]. Cillino et al. used a combination of heavy silicone oil and perfluorohexyloctane (Densiron-68) in 11 eyes with persistent macular holes (mean minimum linear diameter 740.5 µm ± 105.3) and achieved closure in 82% [14]. U-shaped closure (normal foveal contour), i.e., type 1 closure, was seen in seven eyes (64%) [14]. Similarly, Lappas and al. demonstrated Densiron-68-induced closure in 11 of 12 persistent macular holes (mean minimum linear diameter 502.3 µm ± 129.4), although closure patterns were not evaluated [20]. Furthermore, Rizzo et al. reported type 1 closure in 20 of 23 macular holes (mean minimum linear diameter 560 μm, SD n.a.) using Densiron-68, type 2 closure in one of 23 and non-closure in 2/23 eyes [11]. A recent retrospective series found a closure rate of 90.9% in 33 persistent idiopathic macular holes (mean minimum linear diameter 391 μm ± 137.8) using silicone oil (2000-centistoke SO in 30/33 eyes and 5000-centistoke SO in 3/33 eyes), although closure types were not specified [13]. Lohmann and colleagues reported anatomical success in 79.4% of 63 cases treated with heavy silicone oil (Densiron-68 or Oxane-HD, only type 1 closure was counted as anatomical success) [19]. Nowroozzadeh et al. reported types 1 and 2 closure in 69.2% and 15.4% of 13 idiopathic macular holes using light SO, although preoperative macular hole diameters were not reported [12]. Similarly, Kumar et al. used light SO for idiopathic macular holes and reported types 1 and 2 closure in 75% and 25%, respectively, but their sample size was limited to 8 eyes [21]. We conclude that previous studies addressing SO tamponade for refractory macular holes are difficult to compare due to differences in SO type (light vs. heavy silicone oil), inconsistencies in reporting closure patterns and preoperative macular hole diameters, and low sample size. Furthermore, some authors consider flat open holes (type 2 closure) to be open [14], whereas others assume the opposite [20]. In several studies, the closure type is not further specified [13, 20, 22]. Previous investigations that employed light rather than heavy silicone oil are of particular interest for our study results. Unfortunately, the series with the biggest sample size did not specify closure patterns [13], which leaves us to speculate on the rate of eyes exhibiting the favorable type 1 closure pattern. On the other hand, Kumar and Nowroozzadeh showed comparable results to our investigation, but their samples were limited [12, 21].

Major drawbacks of SO tamponade include rare cases of vision loss due to retinal toxicity [23] and the necessity of additional surgery for oil removal, which is not required following other surgical options. Small macular holes constitute a relevant subset of persistent macular holes (5% in our study). Due to their favorable prognosis, less invasive procedures such as gas injection or simple vitrectomy with extended ILM peeling and fluid-air exchange with or without expanding gas tamponade may be beneficial. Other options for larger MHs include re-instillation of conventional gas, implanting a scaffold for glial proliferation (ILM translocation/insertion/free flap, amniotic membrane, lens capsule, retinal tissue), increasing retinal compliance (macular hole hydrodissection/induction of retinal detachment), and stimulation of retinal adhesion (autologous blood, autologous platelet concentrate). A study investigating conventional gas refill previously reported types 1 and 2 closure rates of 62.2%/27.8% (mean minimum linear diameter not given) [15], respectively, which would imply that conventional gas refill is equivalent to the instillation of light SO tamponade. On the other hand, a prospective randomized study previously showed type 1 closure using Densiron-68 (mean minimum linear diameter 740.5 μm ± 105.3) in 82% compared to 30% of cases with gas (mean minimum linear diameter 680.3 μm ± 120.8) [14].

Previous studies investigating autologous ILM flap transplantation in refractory macular holes reported type 1 and type 2 closure rates of 92%/8% (mean minimum linear diameter 1637.6 μm, SD 412.7) [24] and 75%/0% (mean minimum linear diameter 468 μm, SD 106) [25], respectively. Macular hole hydrodissection studies achieved types 1 and 2 closure rates of 83%/0% (mean minimum linear diameter not given) [26] and 67%/8% (mean minimum linear diameter 606 μm, SD 168) [25], respectively. For epiretinal amniotic membrane transplantation, type 1/type 2 closure rates of 62.5%/0% (mean minimum linear diameter 953.5 μm, SD 436) [25] and 71%/29% (mean minimum linear diameter 494 μm ± 210) were reported [27]. Lens capsular flap achieved type 1/type 2 closure rates of 96% / 4% (mean minimum linear diameter 1102 μm, SD 561) [28] and 75%/15% (mean minimum linear diameter 788.80 μm, SD 198.11) [29]. Autologous retinal transplantation attained a type 1 closure rate of 79% (mean minimum linear diameter 796 μm ± 117), although the frequency of type 2 closure was not reported [30]. Generally, reports of these mostly new approaches were limited by small sample sizes and variability of inclusion criteria such as concomitant ocular diseases, the inclusion of myopic MHs and MH sizes. It should furthermore be noted that the instillation of SO does not exclude other techniques per se. Rather, SO represents an alternative to other tamponades (i.e., air/gas). In addition to SO instillation, ILM remnants were managed (additional ILM peeling in nine cases and ILM-flap creation in three cases) in our study, which may have contributed to MH closure. The positive effect of ILM peeling on primary MH closure rates was established by Eckardt et al. in 1997 [31]. Other studies have shown that additional ILM peeling for refractory MH is safe and effective [32, 33]. ILM flap creation is beneficial for MH closure as well [24, 25]. Additionally employed surgical techniques should be controlled in future randomized controlled trials to carve out the actual effects of SO.

Best-corrected visual acuity of our patients improved significantly from 0.99 logMAR before to 0.74 logMAR after SO instillation (*p* = 0.035, *n* = 21). This is in line with the literature, as most studies showed a significant increase in visual acuity after tamponade with light and heavy SO, although high levels of vision were rarely achieved [12–14, 20]. We observed significant BCVA improvement in eyes with type 1 closure, but eyes with type 2 and non-closure showed no improvement.

Limitations of our study include its retrospective design with all its inherent drawbacks, especially missing postoperative BCVA scores in many cases. However, we believe that the stratification for closure types compensates for this limitation to some degree. Patients with type 1 closure can be expected to attain satisfactory visual results.

Strengths of our study include a large sample size and stratification for macular hole closure patterns. To our knowledge, this is the largest investigation of light SO tamponade for persistent macular holes. Because light SO is easier to remove than heavy SO, we believe that light SO should generally be preferred to heavy SO. SO represents an effective treatment approach for patients unable to adhere to postoperative positioning, although face-down positioning is advisable even after SO tamponade and was recommended to all of our patients. Patient compliance in follow-up is urgently required, because SO removal should be attempted in all cases. In our series, one patient refused silicone oil removal. Thus, uncompliant patients who are not able to undergo SO removal should not be offered SO tamponade. In conclusion, SO instillation is a beneficial surgical approach in selected cases of macular holes’ persistence resulting in high anatomic closure rates. The desirable type 1 closure pattern can be expected in approximately 69% of cases and this information should be transparently communicated to patients who require re-vitrectomy.
